# Effect of synthetic CT on dose-derived toxicity predictors for MR-only prostate radiotherapy

**DOI:** 10.1093/bjro/tzae014

**Published:** 2024-06-03

**Authors:** Christopher Thomas, Isabel Dregely, Ilkay Oksuz, Teresa Guerrero Urbano, Tony Greener, Andrew P King, Sally F Barrington

**Affiliations:** School of Biomedical Engineering & Imaging Sciences, King’s College London, SE17EH London, United Kingdom; Medical Physics Department, Guy’s and St Thomas’ Hospital NHS Foundation Trust, SE17EH London, United Kingdom; School of Biomedical Engineering & Imaging Sciences, King’s College London, SE17EH London, United Kingdom; Computer Science, UAS Technikum Wien, 1200 Vienna, Austria; School of Biomedical Engineering & Imaging Sciences, King’s College London, SE17EH London, United Kingdom; Computer Engineering Department, Istanbul Technical University, 34485 Istanbul, Turkey; Clinical Oncology, Guy’s and St Thomas’ Hospital NHS Foundation Trust, SE17EH London, United Kingdom; Medical Physics Department, Guy’s and St Thomas’ Hospital NHS Foundation Trust, SE17EH London, United Kingdom; School of Biomedical Engineering & Imaging Sciences, King’s College London, SE17EH London, United Kingdom; School of Biomedical Engineering & Imaging Sciences, King’s College London, SE17EH London, United Kingdom; King’s College London and Guy’s and St Thomas’ PET Centre, School of Biomedical Engineering and Imaging Sciences, King’s College London, King’s Health Partners, SE17EH London, United Kingdom

**Keywords:** synthetic CT, rectal toxicity, prostate, artificial intelligence, deep learning

## Abstract

**Objectives:**

Toxicity-driven adaptive radiotherapy (RT) is enhanced by the superior soft tissue contrast of magnetic resonance (MR) imaging compared with conventional computed tomography (CT). However, in an MR-only RT pathway synthetic CTs (sCT) are required for dose calculation. This study evaluates 3 sCT approaches for accurate rectal toxicity prediction in prostate RT.

**Methods:**

Thirty-six patients had MR (T2-weighted acquisition optimized for anatomical delineation, and T1-Dixon) with same day standard-of-care planning CT for prostate RT. Multiple sCT were created per patient using bulk density (BD), tissue stratification (TS, from T1-Dixon) and deep-learning (DL) artificial intelligence (AI) (from T2-weighted) approaches for dose distribution calculation and creation of rectal dose volume histograms (DVH) and dose surface maps (DSM) to assess grade-2 (G2) rectal bleeding risk.

**Results:**

Maximum absolute errors using sCT for DVH-based G2 rectal bleeding risk (risk range 1.6% to 6.1%) were 0.6% (BD), 0.3% (TS) and 0.1% (DL). DSM-derived risk prediction errors followed a similar pattern. DL sCT has voxel-wise density generated from T2-weighted MR and improved accuracy for both risk-prediction methods.

**Conclusions:**

DL improves dosimetric and predicted risk calculation accuracy. Both TS and DL methods are clinically suitable for sCT generation in toxicity-guided RT, however, DL offers increased accuracy and offers efficiencies by removing the need for T1-Dixon MR.

**Advances in knowledge:**

This study demonstrates novel insights regarding the effect of sCT on predictive toxicity metrics, demonstrating clear accuracy improvement with increased sCT resolution. Accuracy of toxicity calculation in MR-only RT should be assessed for all treatment sites where dose to critical structures will guide adaptive-RT strategies.

**Clinical trial registration number:**

Patient data were taken from an ethically approved (UK Health Research Authority) clinical trial run at Guy’s and St Thomas’ NHS Foundation Trust. Study Name: MR-simulation in Radiotherapy for Prostate Cancer. ClinicalTrials.gov Identifier: NCT03238170.

## Introduction

Successful radiotherapy (RT) is a balance between irradiating tumour cells adequately for tumour control and limiting dose to healthy tissues. Recently, the integration of PET and MR imaging has allowed the use of functional information to guide RT planning and adaptation.^1–3^ The advent of MR-guided treatment machines^4^ promises to further widen the therapeutic window,^5–7^ by facilitating daily adaptive treatment regimens^8–11^ with increased accuracy afforded by enhanced soft tissue contrast.

The concept of isotoxic adaptive RT has been proposed,^9^^,^^12^^,^^13^ whereby dose prescription and distribution are optimized at each fraction for TCP by maintaining predicted toxicity at a predetermined level. The clinical benefit of adaptive MR-guided treatment delivery is being validated using NTCP models,^14^ and the concept of toxicity-guided adaptive RT has been proposed. For prostate RT in particular, a recent study highlighted the use of toxicity risk models to estimate the benefit of MR-guided adaptive RT^15^ and clinical trials including the MIRAGE trial (NCT04384770) will evaluate MR-guided- against conventional CT-guided RT in terms of gastrointestinal (GI) and genitourinary (GU) toxicity.

Crucial to the success of isotoxic and toxicity-driven RT protocols is accurate visualization of anatomy and accurate dose calculation. By integrating MR imaging into RT treatment machines, MR linear accelerators (MR-linacs)^4^ allow MR imaging at the time of treatment delivery and offer improved anatomical visualization over CT for some tissues. However, in a truly MR-only environment, accurate dose calculation is dependent on correct representation of electron density through the use of synthetic CTs (sCT).^16^ Methods of sCT generation must be accurate enough to maintain dose calculation accuracy to critical structures for decision-making using accurate toxicity estimation.

This first evaluation of the effect of sCT methodology on toxicity risk prediction concentrates on rectal toxicity from prostate RT. In the United Kingdom, 10% of patients receiving prostate RT experience gastrointestinal toxicity within the first 2 years post RT.^17^ The most established method of dose-derived rectal toxicity risk estimation uses Lyman-Kutcher-Burman (LKB) models^18^ where dose volume histogram (DVH) data from the rectum are used. These models have been used as the basis for prostate escalation trials.^19^ In recent years risk modelling has been performed by extracting dose surface maps (DSM) from the rectal wall. Buettner et al^20^ created and parameterized binary DSMs for the rectum and reported associations with rectal toxicity in patients recruited to the RT01 trial [ISRCTN 47772397]. The most statistically significant associations with rectal bleeding were the relative area of the 51 Gy DSM > 0.374 (*P* = .03), and the relative lateral extent of the 61 Gy DSM > 0.591 (*P* < .001).

The aim of this work was to evaluate the effect of sCT methodologies on methods of rectal toxicity prediction using an LKB model based on rectal DVH and a model based on rectal DSM.

## Methodology

### Patient datasets and scan acquisition

Thirty-six patients were recruited into a local Research and Development and ethical-review-board approved clinical study [NCT03238170] registered on clinicaltrials.gov, giving written informed consent. Patients underwent large field of view (LFOV) diagnostic quality T2-weighted and T1 Dixon MR scans within 3 h of their standard-of-care (SoC) RT planning CT scan for prostate RT. All CT scans were acquired with 2.5 mm slice thickness with in-plane pixel dimensions of 0.98 mm. MR scans were acquired on a 1.5 T Aera scanner (Siemens AG, Munich, Germany). The T2 turbo spin echo (T2tse) MR was optimized to provide image quality suitable for accurate delineation of all prostate target volumes and relevant organs at risk (OAR), with TE = 104 ms, TR = 14710 ms, and voxel size 0.4 mm × 0.4 mm × 3 mm. A T1 Dixon sequence was acquired using Siemens standard protocol. Bladder and rectal preparation were replicated for the MR and patients were scanned in the same treatment position using a combination of immobilization equipment supplied by Q-fix and equipment manufactured in-house.

For the DL approach, the 36 patient data sets were split into 24 patients for training and 12 for testing. Treatment plans were created on their planning CT, delivering 60 Gy in 20 fractions according to results from the CHHiP clinical trial (ISRCTN97182923).^21^ The volumetric modulated arc therapy (VMAT) treatment plans were calculated in Eclipse (Varian Oncology Ltd, CA, United States) using Acuros algorithm, calculating dose to medium. For each plan, planning target volume (PTV) doses were prescribed such that dose to 95% (D95%) of PTV60 was ≥57 Gy, with median dose = 60 Gy, and PTV48 D95% ≥ 45.6 Gy. The most recent rectal dose constraints^22^ were applied and met in all cases, and all OAR tolerances were met.

### Synthetic CT datasets

For each test patient, 4 sCTs were generated from the MR data. These are described below.

#### Bulk density approach (sCT_BDw, sCT_BDp)

Bulk density (BD) sCTs were created by the authors by assigning all pixels within the T2tse body surface to Hounsfield Unit (HU) = 0 to generate sCT_BDw, and assigning all body pixels to HU = 11 (population average HU density over the 24 non-test patients) to generate sCT_BDp.

#### Tissue stratification [using siemens synthetic CT product] (sCT_TS)

The commercially available Siemens CE-marked syngo.via VB30 (Siemens AG, Munich, Germany) application for tissue-stratified synthetic CT generation^23^ was used to create sCT_TS from the T1 Dixon images, resulting in a dataset with 4 internal tissue classes (fat, muscle, spongy bone, compact bone) and inside air (e.g. rectal gas). Non-rigid multi-modality mutual-information registration to the planning CT was performed in MIM (MIM Software Inc, OH, United States) to mitigate against the anatomical variation arising from the time delay between CT and MR and the process of repeat patient setup on the MR.

To assess the effect on calculated dose from residual anatomical differences after the non-rigid-registration, we created an extra dataset whereby the ground truth planning CT (rather than the T1 Dixon MRI) was stratified into tissue types to mimic the Siemens tissue stratification (TS) method. This dataset is referred to throughout as sCT_TS(CT). Here, the 4 internal tissue classes (fat, muscle, spongy bone, and compact bone) and air inside the rectum were auto-segmented using a threshold method^24^ on the planning CT and assigned HU according to nominal Siemens sCT_TS values (fat = −75HU, muscle/bladder = 0HU, spongy bone = 204HU, cortical bone = 1067HU, inside air = −500HU). Calcifications and implanted fiducial markers within the clinical target volume (CTV) were assigned as muscle. Areas thresholded as fat or muscle within the spongy bone structures were assigned spongy bone.

#### Deep learning artificial intelligence approach (sCT_AI)

A deep learning (DL) network was trained, by the authors, to generate the fourth sCT: sCT_AI. The 24 non-test patients formed the training dataset and were used to train a convolutional neural network (CNN) to generate sCT from the T2tse MR data. Data preparation for the CNN training and test data was performed within MIM whereby a multi-modality mutual-information non-rigid image registration algorithm deformed the LFOV T2tse MR scans to the patient’s corresponding planning CT scan. The deformed MR data was resampled to CT-resolution in all planes. MR pixel data was normalized from 0 to 1, relative to the near maximum (99.995 percentile) value in the MR. Normalization of the planning CT applied a cut-off HU of 2000, and subsequent normalization using eq (1).

(1) $$CT_{\mathrm{arrayNorm}}=\frac{CT_{\mathrm{array}}}{3000}+\;\frac{1}{3}$$

**Figure 1.**
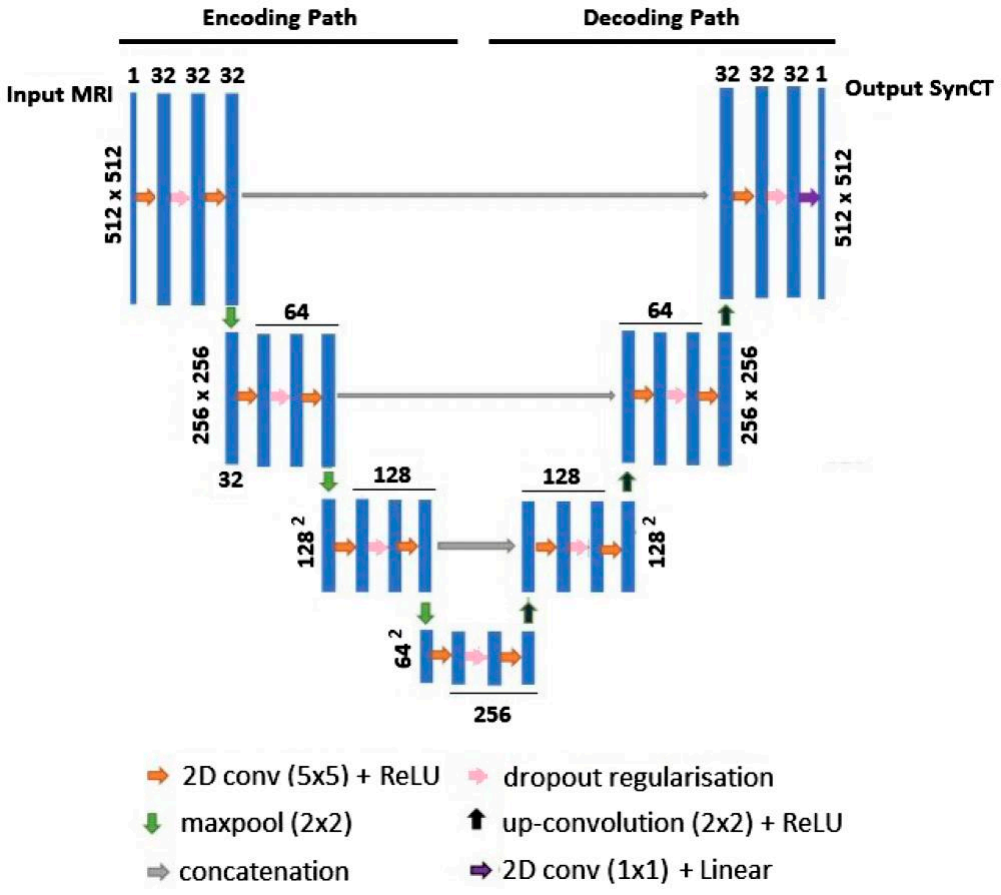
CNN (U-Net) design.

The 2D MR slices now represent the “image” data, with the corresponding 2D CT slices representing the “labels.” Network design and hyper-parameter tuning of number of network levels, kernel size, dropout rate, and number of epochs was performed using 12 of the 24 training patients in a leave one-out cross-validation approach, with 11/12 patients comprising the training set for each fold. Simple data augmentation (translation, flipping, and rotations) provided no improvement in accuracy at this stage. The final architecture consisted of a 2D U-Net^25^ of 32 layers within 4 levels, utilizing dropout regularization rate of 0.2, rectified linear unit (ReLU) activation functions and linear final activation function. The whole batch of training data was input per epoch using the Adam optimizer,^26^ with learning rate 1e-4, mean squared error as loss function, and trained for 100 epochs. The U-Net architecture (Figure 1) was written and refined in Python, within the Keras environment^27^ using Tensorflow^28^ as the backend. Processing was performed on a 12 Gb Titan Xp graphics processing unit (GPU).

Training input data consisted of 2 (512 × 512 × 1671) arrays: one containing 1671 MR slices from the 24 patients and the other containing the corresponding CT slices as training labels.

### Assessment of sCT dose calculations on rectal toxicity risk prediction

The treatment plans originally optimized and calculated on the planning CT were transferred to each sCT dataset and recalculated using identical algorithm and monitor units (MU). Resulting dose distributions were compared against the ground truth CT dose distribution using gamma evaluation^29^ in Verisoft v6.1 (PTW, Freiburg, Germany) software. LKB model parameters (TD_50_ = 97.7 Gy, *m* = 0.27, *n* = 0.085, *a*/*b *= 3 Gy) were used to predict risk of grade 2 rectal bleeding and late faecal incontinence (LFI) (TD_50_ = 105.0 Gy, *m* = 0.43, *n* = 1.0, *a*/*b* = 3 Gy) from DVH data as adopted in the methodology of Onjukka et al.^19^ The LKB methodology is described in the literature^30^ and has been incorporated in commercially available software which allows use of DVHs from the calculated treatment plans for LKB prediction of toxicity risk.^31^ Risk predictions from each sCT were compared against those calculated from the ground truth planning CT.

DSMs were extracted for each of the dose distributions following the methodology outlined by Buettner et al.^20^ On each axial slice, the posterior-most point of the rectal contour was located and, moving clockwise around the contour, the dose to each pixel on the rectal surface input into an array. This was repeated for each axial slice of the rectum. The Buettner paper proceeded to interpolate this array to a 21 × 21 array to compare DSMs between patients. Buettner et al worked with 5 mm CT slice separation, whereas data from this study has 2.5 mm separation, therefore, interpolation within this work is to a 42 × 42 array, with 42 elements in the cranio-caudal direction and 42 in the circumferential direction to maintain a square DSM. The DSMs were converted to equivalent dose in 2 Gray fractions (EQD2) using an alpha-beta ratio of 3. From each DSM, binary maps were created for 51 and 61 Gy. The area of the 51 Gy region relative to the full DSM area was found. An ellipse was fitted to the largest cluster of voxels within each 61 Gy binary DSM. Ellipse parameters were exported and length of principal lateral axis of the ellipse recorded. All DSM parameters from sCT dose calculations were evaluated against those from the ground truth planning CT.

## Results

### Synthetic CT datasets

**Figure 2.**
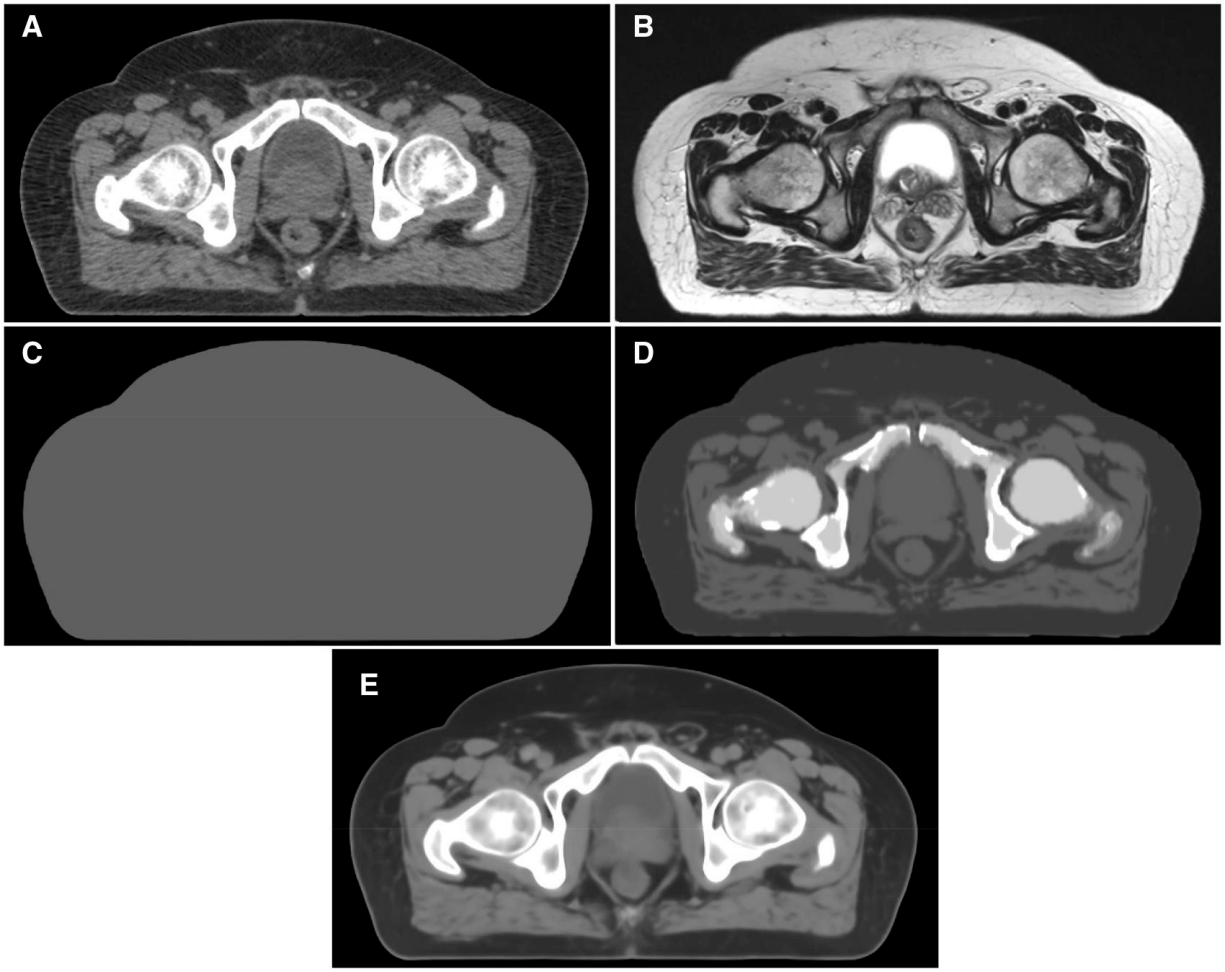
Axial slice from representative patient showing (A) planning CT, (B) T2tse, (C) sCT_BDw, (D) sCT_TS, and (E) sCT_AI.

Four sCT datasets were generated for each patient using the 3 strategies of BD, TS, and DL. The increasing level of HU intensity resolution is illustrated in Figure 2. The sCT_AI datasets have voxel-wise HU resolution that delivers increased accuracy and detail in the bony anatomy, at the prostate/bladder interface and within the rectum compared to sCT_TS.

Training of the CNN on 24 patients reached a plateau after 100 epochs and took 5 h. At inference, generation of sCT_AI for each test dataset took less than 3 s. The mean error (ME) and mean absolute error (MAE) in HU within the body volume over all patients was −2.8 ± 2.4HU (one SD) and 36.8 ± 2.4HU respectively (Table 1). Mean SD of HU error over the body volume was 87.7 ± 7.1HU.

**Table 1.** sCT_AI HU analysis accuracy against planning CT scan for 12 test patients.

sCT_AI dataset	ME	SD of	MAE
	(HU)	HU errors (HU)	(HU)
1	−0.8	94.7	37.6
2	−3.3	102.9	40.5
3	0.5	84.3	36.1
4	−5.8	79.2	33.4
5	−5.7	87.5	37.2
6	−4.5	88.3	35.9
7	−1.4	95.4	40.6
8	−5.1	86.0	35.0
9	−2.6	89.0	40.1
10	−1.0	81.0	35.0
11	1.3	84.5	35.0
12	−4.6	79.2	35.6
Mean	−2.8	87.7	36.8
SD	2.4	7.1	2.4

### Assessment of sCT dose calculations on rectal toxicity risk prediction

#### Dose distribution and DVH analysis

All sCT dose distributions passed 3D local gamma analysis with >95% of pixels passing at 2%/2 mm (evaluated over points receiving >20% of max dose). For PTV60 median dose, mean error ± SD were 0.9  ± 0.2, 0.6 ± 0.2, 0.6 ± 0.2, and 0.0 ± 0.1 Gy for sCT_BDw, sCT_BDp, sCT_TS, and sCT_AI respectively. Maximum absolute errors in rectal DVH parameters were 1.1%, 0.9%, 0.9%, and 0.4% for sCT_BDw, sCT_BDp, sCT_TS, and sCT_AI respectively with median errors 0.5%, 0.3%, 0.5%, and 0.1%. For 12 treatment plans originally passing the V60Gy < 0.0% rectal constraint when calculated using the patients’ planning CT scans, 10 failed the constraint using a BD approach, 9 using the TS approach and 1 using sCT_AI.

#### LKB-derived late faecal incontinence risk

Ground truth predicted late faecal incontinence risk ranged between 2.1% and 4.0%, with median risk 3.2%. The ground truth range, alongside a box plot of absolute errors introduced for each sCT method, is shown in Figure S1. Despite each BD and TS sCT resulting in statistically significant errors in risk calculation compared to ground truth (Table S1), the maximum error in LFI risk prediction for all sCT methodologies was a clinically insignificant 0.1%. sCT_AI resulted in accurate LFI risk calculation in all cases.

#### LKB-derived G2 rectal bleeding risk

**Figure 3.**
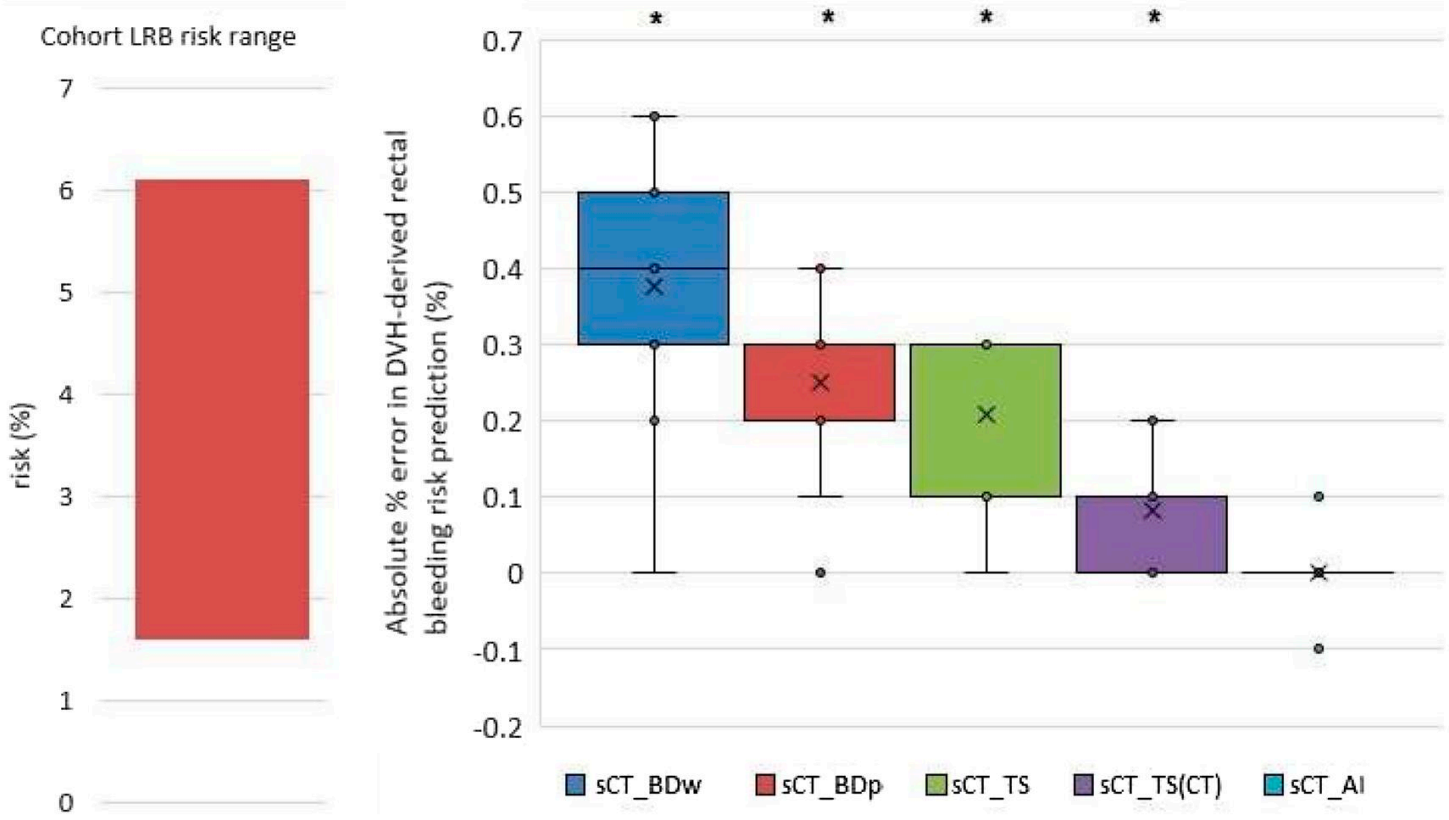
Left: Range of ground truth G2 LRB risk predictions for the 12 test patients, Right: Error in risk prediction due to sCT method. Boxes represent the interquartile range (IQR). Whiskers represent the largest value within 1.5 times the IQR above and below the box. Mean value is shown as a cross. Statistical significance (paired *t*-test, *P* < .05) indicated by asterisk, after normal distribution was confirmed by Shapiro-Wilk test.

Ground truth predicted G2 rectal bleeding risk ranged between 1.6% and 6.1%, with median risk 4.0%. Figure 3 shows this ground truth range alongside a box plot of absolute errors introduced for each sCT method.

Dose distributions calculated on sCT_BDw gave the largest deviations to DVH-based LKB-derived risk, with lower and upper 95% agreement limits of 0.04% and 0.71% respectively (Table 2). sCT_BDp addressed some of this bias by assigning a more appropriate population-based uniform density. sCT_TS reduced the mean error and 95% agreement limits further, albeit still with statistically significant errors compared with the gold standard. Simulating the TS approach on the planning CT (sCT_TS(CT)) highlighted the effect of unresolved registration errors with the T1-Dixon, bringing the mean error down by 0.13%. sCT_AI dose calculations delivered statistically similar LKB risk predictions to dose calculation on the gold standard CT, with maximum absolute errors of 0.1% and 95% agreement limits of ±0.12%.

**Table 2.** Error statistics for G2 LRB risk predictions derived from each sCT.

Error in DVH-derived G2 rectal bleeding risk prediction	sCT_BDw	sCT_BDp	sCT_TS	sCT_TS(CT)	sCT_AI
Mean absolute error (absolute %)	0.38	0.25	0.21	0.08	0.00
SD (absolute %)	0.17	0.12	0.12	0.07	0.06
Upper 95% acceptance limit (absolute %)	0.71	0.48	0.44	0.22	0.12
Lower 95% acceptance limit (absolute %)	0.04	0.02	−0.02	−0.06	−0.12
*P*-value (paired *t*-test)	<.001	<.001	<.001	.002	1.000

#### DSM-derived G2 rectal bleeding risk

**Figure 4.**
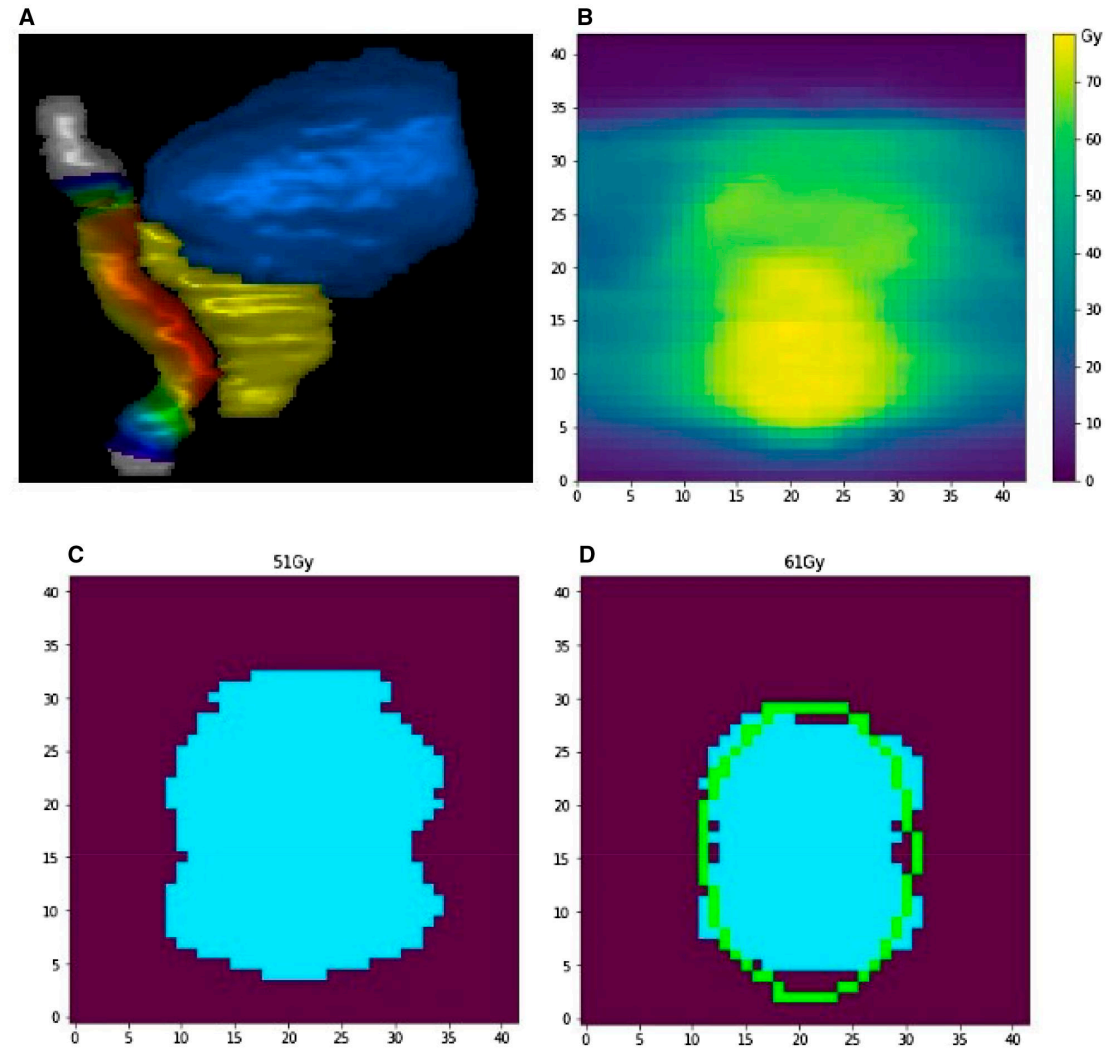
DSM methodology and results for representative patient. (A) Surface dose wash displayed on 3D render of rectum [grey (online version only)] adjacent to clinical target volumes [yellow (online version only)] and bladder [blue (online version only)], (B) EQD2-corrected rectal DSM, (C) binary map of EQD2 dose > 51 Gy, (D) binary map of EQD2 dose > 61 Gy with superimposed ellipse [green (online version only)].

Figure 4 demonstrates the DSM methodology for a representative patient, highlighting how the rectal surface dose distribution (4a) maps to a rectal DSM (4b) and displays the resulting binary maps (4c and 4d).

**Figure 5.**
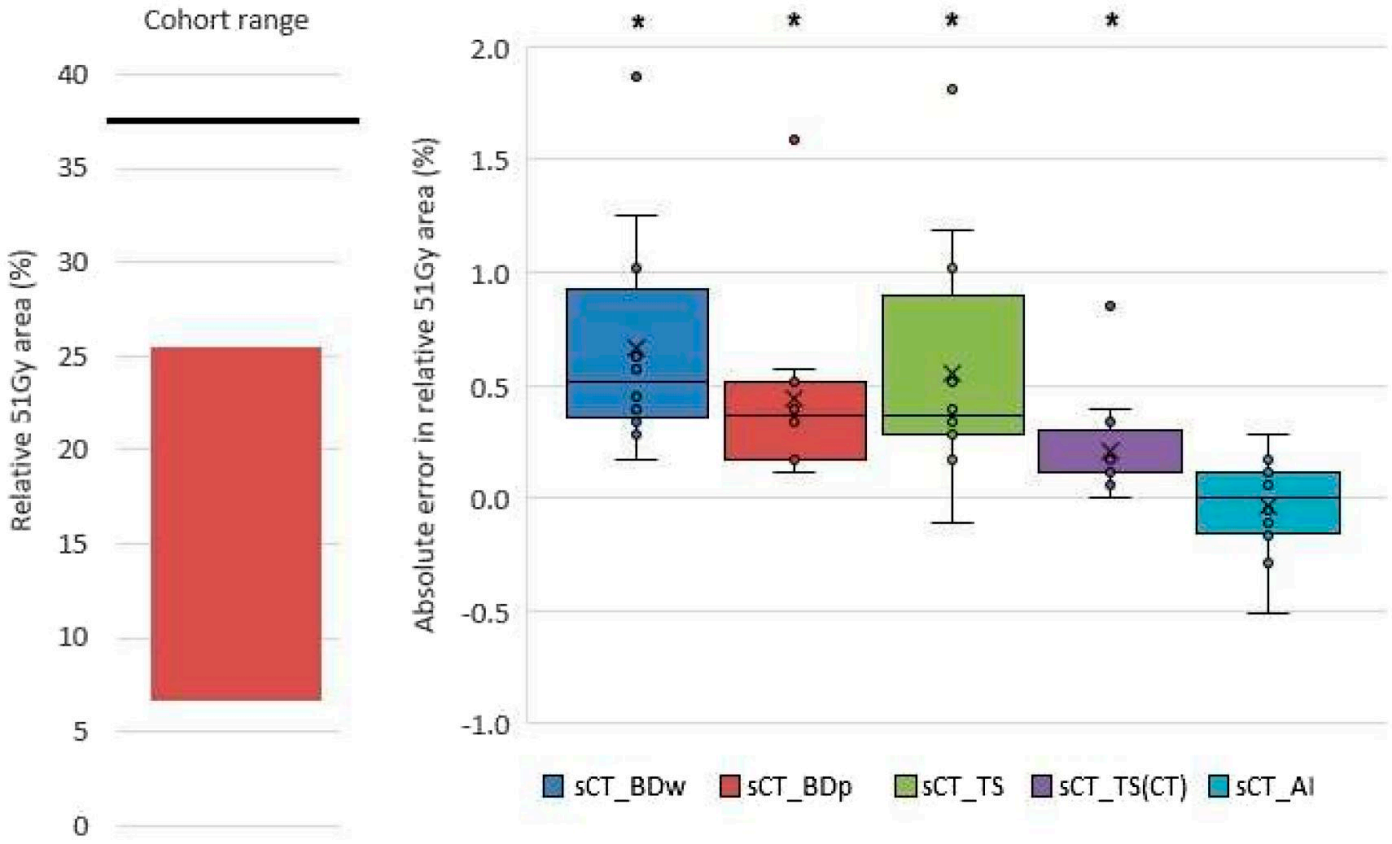
Left: test cohort range of 51 Gy relative area on DSM with 37.4% tolerance indicated as thick horizontal line. Right: absolute errors introduced by sCT. Boxes represent the IQR. Whiskers represent the largest value within 1.5 times the IQR above and below the box. Mean (cross) and median (line) are also shown. Statistical significance (paired *t*-test, *P* < .05) indicated by asterisk, after normal distribution was confirmed by Shapiro-Wilk test.

Ground truth relative 51 Gy area of DSM ranged between 6.6% and 25.5% with median 17.0%. All ground truth plans resulted in values less than the 37.4% tolerance. Figure 5 shows this ground truth range alongside a box plot of absolute errors introduced for each sCT method.

**Figure 6.**
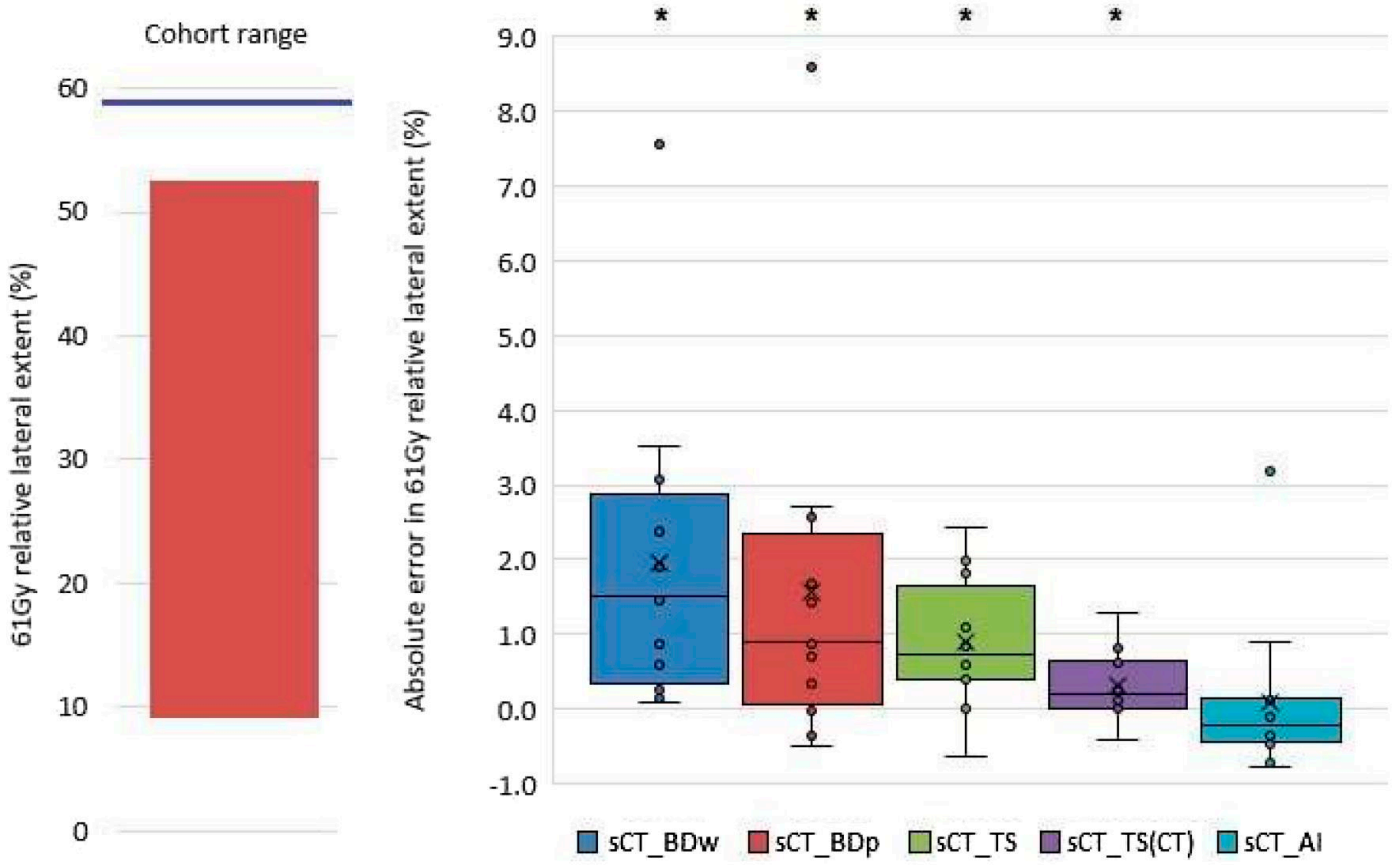
Left: test cohort range of 61 Gy relative lateral extent on DSM with 59.1% tolerance indicated as thick horizontal line. Right: absolute errors introduced by sCT. Boxes represent the IQR. Whiskers represent the largest value within 1.5 times the IQR above and below the box. Mean (cross) and median (line) are also shown. Statistical significance (paired *t*-test, *P* < .05) indicated by asterisk, after normal distribution was confirmed by Shapiro-Wilk test.

Ground truth relative lateral extent of 61 Gy in the DSMs ranged between 9.1% and 52.5% with median 34.3%. All ground truth plans resulted in values less than the 59.1% tolerance. Figure 6 shows this ground truth range alongside a box plot of absolute errors introduced for each sCT method.

## Discussion

sCT accuracy for prostate RT planning in an MR-only pathway has been reported,^16^ with many authors establishing deep learning methods for voxel-wise sCT generation from MR.^32^ Analysis of errors has focussed primarily on treatment planning parameters, reporting errors in target and OAR doses. Our work evaluates the effect of sCT inaccuracies on conventional treatment planning and importantly on the risk prediction of normal tissue toxicity, in this case G2 rectal bleeding. As RT strategies move to toxicity-driven adaptive regimes, it is important to assess the effect of sCT on the toxicity predictions that direct them. Our DL model generated sCTs with mean HU error (−3 ± 2HU) comparable to Maspero et al^33^ who reported a mean error of 1 ± 6HU using a similar 2D technique. The mean absolute error for our 12 test patients of 36.8 ± 2.4HU also compares favourably with similar published machine-learning methods, with MAE of 43.3 ± 2.9 HU reported by Bragman et al,^34^ 40.5 ± 5.4HU by Jie Fu et al,^35^ 65 ± 10HU by Maspero et al,^33^ and MAE of 42.4HU in a recent literature review of DL sCT approaches.^32^ Even greater accuracy and robustness may be achieved by increasing the number of training cases, including larger variation in patient size and internal anatomy characteristics, performing data augmentation during training and utilization of more recent CNN architectures. Model performance may also improve with the use of alternative deformable registration algorithms that resolve more of the anatomical differences between CT and MR in the training dataset. Model performance stated above is reliant on high image quality CT images acquired for RT planning, and only for patients scanned head-first supine and on a flat couch-top.

Acceptable accuracy for radiotherapy treatment planning is often considered to be within 2%,^36^ and thus the high gamma pass rates assessed at 2%/2 mm indicate that all sCT methods performed well globally. Global assessment of HU and dose distribution as described above do not, however, assess local errors and the effect on rectal dose calculations and rectal toxicity predictions in particular. In terms of rectal dose parameters used for treatment planning, sCT_BDw produced a noticeable bias in the dosimetric results and in accordance with the study by Kim et al^37^ this bias was reduced when using a uniform density derived from the patient population (sCT_BDp). Errors in PTV and rectal dose constraints agreed closely with the 1% dose uncertainties quoted for pelvic regions in a recent literature review of recent DL sCT approaches^32^ and 1% uncertainties cited in a literature review of atlas and non-DL voxel-based approaches.^16^

In relation to rectal toxicity risk prediction, the DVH-based LKB results demonstrated higher inaccuracies for LRB toxicities than LFI toxicities, indicating that sCT methodology primarily affects the higher doses in the rectal DVH rather than the whole DVH. The voxel-based sCT_AI had the lowest deviation and lowest bias of all methodologies for rectal toxicity risk prediction calculated from rectal DVH, and errors were statistically insignificant when compared with ground truth calculations performed on gold standard planning CT. Bulk density approaches and the TS approach showed statistically significant differences in both DVH- and DSM-derived toxicity metrics. With DSM-based toxicity predictions sCT_AI had superior accuracy compared with the BD and TS techniques, but in this test cohort all sCT dose distributions gave DSM metrics that maintained their position below the cut-off threshold. Despite larger, and statistically significant, errors in rectal toxicity risk calculation for sCT_TS compared to sCT_AI, the level of error for both could be considered clinically appropriate to accurately resolve G2 LRB risk using these risk models. There are numerous other factors that contribute to dose-derived toxicity prediction (such as the accuracy of NTCP models, anatomical delineation and treatment delivery), but it is encouraging that TS- and DL-based approaches both maintain sufficient accuracy for sCT generation in the treatment planning component of the MR-only RT pathway.

Dose calculations performed on sCT_TS and sCT_AI include inherent uncertainty arising from the time between CT and MR acquisition and repeat patient preparation and positioning. Despite best efforts to replicate patient setup and bowel/bladder preparation for the MR scan, changes in internal anatomy could be seen in a few patients which were not completely resolved using deformable image registration. The DL method was robust to these small unresolved registration uncertainties, giving maximum error of 0.1% in DVH-based toxicity. The creation of sCT_TS(CT) allowed inaccuracies originating from residual internal anatomy differences post deformable registration to be quantified for the TS method. The mean error was approximately halved for all toxicity metrics analysed and whilst the errors still remained statistically significant for this idealized TS sCT they would be clinically insignificant for RT purposes.

Despite the limitation of a small test dataset, the study size was sufficient to show clear difference in accuracy between sCT methods. For investigating the use of sCT in non-pelvic sites, where tissue inhomogeneity plays a larger role, larger test datasets would be warranted. Another limitation is the use of DSM tolerances based on conformal treatment planning in the RT01 clinical trial rather than current IMRT planning. IMRT planning within our study meant that all rectal DSM parameters analysed were well within tolerance. However, we detected only very minor DSM differences with the commercial TS and DL approaches, with errors unlikely to affect any future DSM tolerances published based on IMRT delivery.

As well as the increased accuracy for rectal toxicity risk calculation, the voxel-based DL technique has several additional advantages over a stratification method including increased HU resolution, which may be useful for image verification using digitally reconstructed radiographs or cone-beam CT. The DL process requires only the T2 image, meaning a single MR scan can be acquired and utilized for both delineation of target and healthy tissues as well as dose calculation via accurate sCT. There is no requirement for additional T1 Dixon sequence as for the TS process. This provides a crucial time saving in terms of patient throughput.

## Conclusion

Within the RT community there is an increased interest in isotoxic or toxicity-guided regimes, where an assessment of the dose-derived toxicity risk for the patient is used to adapt RT prescription or delivery. It is important to maintain accuracy of toxicity risk estimation when moving to MR-only RT and this work evaluated this with a variety of sCT methodologies. Bulk density approaches introduced significant error in both DVH- and DSM-derived toxicity risk estimation. The more detailed TS and deep learning sCT approaches reduced errors to a clinically appropriate level. It was noticeable that despite the DL voxel-based approach delivering increased HU resolution it did not, in the pelvic region at least, offer clinically significant improvements in DSM- or DVH-based toxicity estimation beyond a tissue-stratification approach. The DL approach was, however, beneficial in other ways. The DL approach required only the T2-weighted sequence for target and OAR delineation, meaning all the treatment planning processes could be carried out using a single MR acquisition, rather than requiring an additional T1 Dixon scan for TS sCT approaches. The speed of DL GPU processing was encouraging, with sCT generated from MR data in seconds.

The assessment of sCT-derived errors should be expanded to other treatment sites where dose to critical structures could guide dose escalation and adaptive radiotherapy strategies. Examples include advanced non-small-cell lung cancer isotoxic escalation, head and neck (H&N) isotoxic escalation and abdominal adaptive stereotactic treatments. sCT accuracy in H&N, thorax, and abdominal regions are less well developed and will be prone to more errors on account of increased tissue heterogeneity. An understanding of the errors introduced into toxicity prediction is necessary before proceeding with MR-only toxicity-guided RT.

## Supplementary Material

tzae014_Supplementary_Data

## Notes

**Senior author:** Sally F. Barrington

## References

[1] Thorwarth D. Biologically adapted radiation therapy. Z Med Phys. 2018;28(3):177-183. 28869163 10.1016/j.zemedi.2017.08.001

[2] Verma V , Choi JI, Sawant A, et al Use of PET and other functional imaging to guide target delineation in radiation oncology. Semin Radiat Oncol. 2018;28(3):171-177. 29933876 10.1016/j.semradonc.2018.02.001

[3] Thorwarth D. Functional imaging for radiotherapy treatment planning: current status and future directions—a review. Br J Radiol. 2015;88(1051):20150056. 25827209 10.1259/bjr.20150056 PMC4628531

[4] Liney GP , Whelan B, Oborn B, Barton M, Keall P. MRI-linear accelerator radiotherapy systems. Clin Oncol. 2018;30(11):686-691. 10.1016/j.clon.2018.08.003 30195605

[5] Datta A , Aznar MC, Dubec M, Parker GJM, O’Connor JPB. Delivering functional imaging on the MRI-Linac: current challenges and potential solutions. Clin Oncol. 2018;30(11):702-710. 10.1016/j.clon.2018.08.005 30224203

[6] Hunt A , Hansen VN, Oelfke U, Nill S, Hafeez S. Adaptive radiotherapy enabled by MRI guidance. Clin Oncol. 2018;30(11):711-719. 10.1016/j.clon.2018.08.001 30201276

[7] Pollard JM , Wen Z, Sadagopan R, Wang J. The future of image-guided radiotherapy will be MR guided. Br J Radiol. 2017;90(February):20160667. 28256898 10.1259/bjr.20160667 PMC5605101

[8] Pathmanathan AU , Van As NJ, Kerkmeijer LGW, et al Magnetic resonance imaging-guided adaptive radiation therapy: a “game changer” for prostate treatment? Radiat Oncol Biol. 2018;100(2):361-373. 10.1016/j.ijrobp.2017.10.020 29353654

[9] Henke L , Kashani R, Robinson C, et al Phase I trial of stereotactic MR-guided online adaptive radiation therapy (SMART) for the treatment of oligometastatic or unresectable primary malignancies of the abdomen. Radiother Oncol. 2018;126(3):519-526. 29277446 10.1016/j.radonc.2017.11.032

[10] Bohoudi O , Bruynzeel AME, Senan S, et al MRI-guided radiotherapy Fast and robust online adaptive planning in stereotactic MR-guided adaptive radiation therapy (SMART) for pancreatic cancer. Radiother Oncol. 2017;125:439-444. 28811038 10.1016/j.radonc.2017.07.028

[11] McPartlin AJ , Li XA, Kershaw LE, et al; MR-Linac Consortium. MRI-guided prostate adaptive radiotherapy—a systematic review. Radiother Oncol. 2016;119(3):371-380. 27162159 10.1016/j.radonc.2016.04.014

[12] Zindler JD , Thomas CR, Hahn SM, Hoffmann AL, Troost EGC, Lambin P. Increasing the therapeutic ratio of stereotactic ablative radiotherapy by individualized isotoxic dose prescription. J Natl Cancer Inst. 2016;108(2):1-6. 10.1093/jnci/djv305 26476075

[13] Murray L , Lilley J, Thompson CM, et al Isotoxic simultaneous integrated boost to dominant intraprostatic lesions using stereotactic ablative radiation therapy and volumetric modulated arc therapy. Int J Radiat Oncol. 2013;87(2):S14-S1 5. 10.1016/j.ijrobp.2014.01.042 PMC4018668 24685447

[14] Chen H , Schneiders FL, Bruynzeel AME, et al Impact of daily plan adaptation on organ-at-risk normal tissue complication probability for adrenal lesions undergoing stereotactic ablative radiation therapy: NTCP advantages of adaptive MR-guided adrenal SABR. Radiother Oncol. 2021;163:14-20. 34343546 10.1016/j.radonc.2021.07.026

[15] Christiansen RL , Dysager L, Hansen CR, et al Online adaptive radiotherapy potentially reduces toxicity for high-risk prostate cancer treatment. Radiother Oncol. 2022;167:165-171. 34923034 10.1016/j.radonc.2021.12.013

[16] Johnstone E , Wyatt JJ, Henry AM, et al Systematic review of synthetic computed tomography generation methodologies for use in magnetic resonance imaging–only radiation therapy. Int J Radiat Oncol Biol Phys. 2018;100(1):199-217. 29254773 10.1016/j.ijrobp.2017.08.043

[17] NPCA. Annual Report 2019 Results of the NPCA Prospective Audit in England and Wales for men diagnosed from April 1, 2017 to March 31, 2018 (published January 2020); 2019:1-46.

[18] Michalski JM , Gay H, Jackson A, Tucker SL, Deasy JO. Radiation dose-volume effects in radiation-induced rectal injury. Int J Radiat Oncol Biol Phys. 2010;76(Suppl. 3):S123-S129. 20171506 10.1016/j.ijrobp.2009.03.078 PMC3319467

[19] Onjukka E , Uzan J, Baker C, Howard L, Nahum A, Syndikus I. Twenty fraction prostate radiotherapy with intra-prostatic boost: results of a pilot study. Clin Oncol. 2017;29(1):6-14. 10.1016/j.clon.2016.09.009 27692920

[20] Buettner F , Gulliford SL, Webb S, Sydes MR, Dearnaley DP, Partridge M. Assessing correlations between the spatial distribution of the dose to the rectal wall and late rectal toxicity after prostate radiotherapy: an analysis of data from the MRC RT01 trial (ISRCTN 47772397). Phys Med Biol. 2009;54(21):6535-6548. 19826203 10.1088/0031-9155/54/21/006

[21] Dearnaley D , Syndikus I, Mossop H, CHHiP Investigators, et al Conventional versus hypofractionated high-dose intensity-modulated radiotherapy for prostate cancer: 5-year outcomes of the randomised, non-inferiority, phase 3 CHHiP trial. Lancet Oncol. 2016;17(8):1047-1060. 27339115 10.1016/S1470-2045(16)30102-4 PMC4961874

[22] Wilkins A , Naismith O, Brand D, CHHiP Trial Management Group, et al Derivation of dose/volume constraints for the anorectum from clinician- and patient-reported outcomes in the CHHiP trial of radiation therapy fractionation. Int J Radiat Oncol Biol Phys. 2020;106(5):928-938. 31987974 10.1016/j.ijrobp.2020.01.003

[23] Healthineers S. MR-only RT planning for the brain and pelvis with Synthetic CT. Published by Siemens Healthcare GmbH · 8026 0919 online · ©Siemens Healthcare GmbH, 2019. Accessed May 16, 2022. https://cdn0.scrvt.com/39b415fb07de4d9656c7b516d8e2d907/1800000006768945/1ed4126c4f76/Whitepaper-MR-only-RT-planning-for-the-brain-and-pelvis-with-synthetic.CT_1800000006768945.pdf

[24] Maspero M , Seevinck PR, Schubert G, et al Quantification of confounding factors in MRI-based dose calculations as applied to prostate IMRT. Phys Med Biol. 2017;62(3):948-965. 28076338 10.1088/1361-6560/aa4fe7

[25] Ronneberger O , Fischer P, Brox T. U-Net: convolutional networks for biomedical image segmentation. MICCAI. 2015;9351:234-241.

[26] Kingma DP , Ba J. Adam: a method for stochastic optimization. Vol. arXiv:1412, ICLR Conference 2015, http://arxiv.org/abs/1412.6980, 2015, preprint: not peer reviewed.

[27] Keras CF. Keras. GitHub, 2015. https://github.com/fchollet/keras

[28] Abadi M , Agarwal A, Barham P, et al TensorFlow: large-scale machine learning on heterogeneous distributed systems. arxiv:160304467, http://arxiv.org/abs/1603.04467, 2016, preprint: not peer reviewed.

[29] Low DA , Harms WB, Mutic S, Purdy JA. A technique for the quantitative evaluation of dose distributions. Med Phys. 1998;25(5):656-661. 9608475 10.1118/1.598248

[30] Sanchez-Nieto B , Nahum AE. Bioplan: software for the biological evaluation of radiotherapy treatment plans. Med Dosim. 2000;25(2):71-76. 10856684 10.1016/s0958-3947(00)00031-5

[31] Uzan J , EswarVee C, Malik Z, Nahum AE. Biosuite, new software for radiobiological customisation of dose and fracton size in Ebrt. Radiother Oncol. 2009;92(Suppl. 1):S239.

[32] Boulanger M , Nunes J-C, Chourak H, et al Deep learning methods to generate synthetic CT from MRI in radiotherapy: a literature review. Phys Medica. 2021;89(February):265-281. 10.1016/j.ejmp.2021.07.027 34474325

[33] Maspero M , Savenije MHF, Dinkla AM, et al Dose evaluation of fast synthetic-CT generation using a generative adversarial network for general pelvis MR-only radiotherapy. Phys Med Biol. 2018;63(18):185001. 30109989 10.1088/1361-6560/aada6d

[34] Bragman FJS , Tanno R, Eaton-Rosen Z, et al Uncertainty in multitask learning: joint representations for probabilistic MR-only radiotherapy planning. Medical Image Computing and Computer Assisted Intervention—MICCAI 2018. MICCAI 2018. Lecture Notes in Computer Science, vol 11073. Springer, Cham; 2018.

[35] Fu J , Yang Y, Singhrao K, et al Deep learning approaches using 2D and 3D convolutional neural networks for generating male pelvic synthetic computed tomography from magnetic resonance imaging. Med Phys. 2019;46(9):3788-3798. 31220353 10.1002/mp.13672

[36] Venselaar J , Welleweerd H, Mijnheer B. Tolerances for the accuracy of photon beam dose calculations of treatment planning systems. Radiother Oncol. 2001;60(2):191-201. 11439214 10.1016/s0167-8140(01)00377-2

[37] Kim J , Garbarino K, Schultz L, et al Dosimetric evaluation of synthetic CT relative to bulk density assignment-based magnetic resonance-only approaches for prostate radiotherapy. Radiat Oncol. 2015;10:239-248. 26597251 10.1186/s13014-015-0549-7 PMC4657299

