# Supplementary material for: Effect of synthetic CT on dose-derived toxicity predictors for MR-only prostate radiotherapy
Source: BJR Open. 2024 Jun 3;6(1):tzae014. doi: 10.1093/bjro/tzae014 (PMC11213647; doi:10.1093/bjro/tzae014)
Supplement: tzae014_Supplementary_Data [file tzae014_supplementary_data.zip › Supplementary Material_Table_S1.docx]

| Error in DVH-derived late faecal incontinence risk prediction | sCT_BDw | sCT_BDp | sCT_TS | sCT_TS(CT) | sCT_AI |
| --- | --- | --- | --- | --- | --- |
| mean absolute error (absolute %) | 0.07 | 0.05 | 0.05 | 0.04 | 0.00 |
| standard deviation (absolute %) | 0.05 | 0.05 | 0.05 | 0.05 | 0.00 |
| upper 95% acceptance limit (absolute %) | 0.16 | 0.15 | 0.15 | 0.14 | 0.00 |
| lower 95% acceptance limit (absolute %) | -0.03 | -0.05 | -0.05 | -0.06 | 0.00 |
| p-value (paired T-test) | < 0.001 | 0.007 | 0.007 | 0.017 | 1.000 |
