# Supplementary material for: Effect of synthetic CT on dose-derived toxicity predictors for MR-only prostate radiotherapy
Source: BJR Open. 2024 Jun 3;6(1):tzae014. doi: 10.1093/bjro/tzae014 (PMC11213647; doi:10.1093/bjro/tzae014)
Supplement: tzae014_Supplementary_Data [file tzae014_supplementary_data.zip › Supplementary Material.docx]

**Supplementary Material**


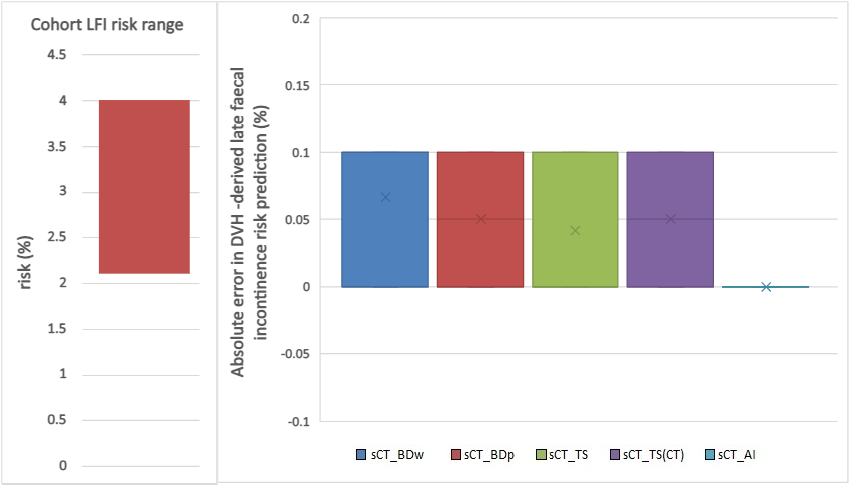


Figure S1 – Left: range of ground truth LFI risk predictions for the 12 test patients, Right: Error in risk prediction due to sCT method. Boxes represent the interquartile range (IQR). Whiskers represent the largest value within 1.5 times the IQR above and below the box. Mean value is shown as a cross. Statistical significance (paired t-test, p < 0.05) indicated by asterisk, after normal distribution was confirmed by Shapiro-Wilk test.

| Error in DVH-derived late faecal incontinence risk prediction | sCT_BDw | sCT_BDp | sCT_TS | sCT_TS(CT) | sCT_AI |
| --- | --- | --- | --- | --- | --- |
| mean absolute error (absolute %) | 0.07 | 0.05 | 0.05 | 0.04 | 0.00 |
| standard deviation (absolute %) | 0.05 | 0.05 | 0.05 | 0.05 | 0.00 |
| upper 95% acceptance limit (absolute %) | 0.16 | 0.15 | 0.15 | 0.14 | 0.00 |
| lower 95% acceptance limit (absolute %) | -0.03 | -0.05 | -0.05 | -0.06 | 0.00 |
| p-value (paired T-test) | < 0.001 | 0.007 | 0.007 | 0.017 | 1.000 |

Table S1 – Error statistics for LFI risk predictions derived from each sCT
