# Supplementary figures and images for: Effect of synthetic CT on dose-derived toxicity predictors for MR-only prostate radiotherapy
Source: BJR Open. 2024 Jun 3;6(1):tzae014. doi: 10.1093/bjro/tzae014 (PMC11213647; doi:10.1093/bjro/tzae014)

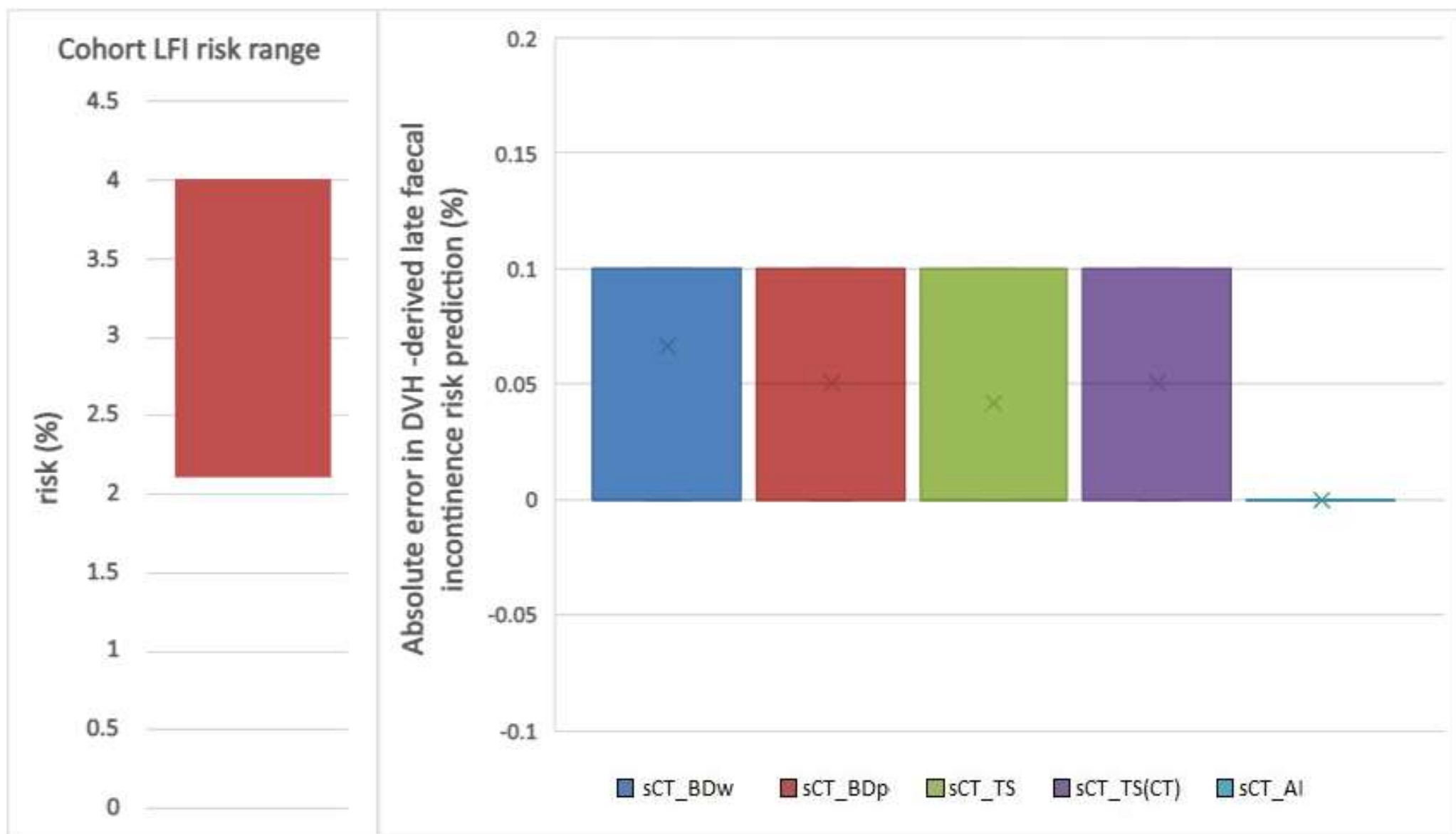

Supplement: tzae014_Supplementary_Data [file tzae014_supplementary_data.zip › Figure S1_NEW.pdf]
